# Nonlinear dynamics and instability of aqueous dissolution of silicate glasses and minerals

**DOI:** 10.1038/srep30256

**Published:** 2016-07-22

**Authors:** Yifeng Wang, Carlos F. Jove-Colon, Kristopher L. Kuhlman

**Affiliations:** 1 Sandia National Laboratories, P. O. Box 5800, Albuquerque, New Mexico 87185-0779, USA

## Abstract

Aqueous dissolution of silicate glasses and minerals plays a critical role in global biogeochemical cycles and climate evolution. The reactivity of these materials is also important to numerous engineering applications including nuclear waste disposal. The dissolution process has long been considered to be controlled by a leached surface layer in which cations in the silicate framework are gradually leached out and replaced by protons from the solution. This view has recently been challenged by observations of extremely sharp corrosion fronts and oscillatory zonings in altered rims of the materials, suggesting that corrosion of these materials may proceed directly through congruent dissolution followed by secondary mineral precipitation. Here we show that complex silicate material dissolution behaviors can emerge from a simple positive feedback between dissolution-induced cation release and cation-enhanced dissolution kinetics. This self-accelerating mechanism enables a systematic prediction of the occurrence of sharp dissolution fronts (vs. leached surface layers), oscillatory dissolution behaviors and multiple stages of glass dissolution (in particular the alteration resumption at a late stage of a corrosion process). Our work provides a new perspective for predicting long-term silicate weathering rates in actual geochemical systems and developing durable silicate materials for various engineering applications.

Chemical weathering of silicate glasses and minerals plays a critical role in global biogeochemical cycles and climate evolution^1^,^2^. Silicate materials have also been used for numerous industrial, biomedical and environmental applications^3^,^4^. Borosilicate glasses have been proposed as a waste form for nuclear waste disposal, and the durability of these materials is a key physical property that needs to be evaluated for waste isolation^4^. Despite decades of intensive research, the mechanism controlling aqueous dissolution of these materials still remains controversial^4^,^5^,^6^,^7^. The debate has centered on the possible formation of a leached surface layer and its role in material dissolution. A silica-rich surface layer has been detected on both manufactured and natural silicate materials^5^,^8^,^9^. Alkali and alkaline cations in this layer are partially leached out and replaced by hydrogen ions during dissolution through a coupled diffusion-ion exchange process. The outer part of the leached layer may continuously be subjected to *in-situ* silicate network repolymerization and reorganization, leading to the formation of a dense silica gel layer that may passivate a dissolving solid surface and result in a dramatic drop in the dissolution rate^3^,^6^. However, this long-held view has recently been challenged by observations of extremely sharp interfaces between altered rims and pristine material domains^7^,^10^,^11^, suggesting that material corrosion may undergo a direct dissolution-precipitation process^7^,^10^. This argument is supported by isotope studies. Isotopes such as ^18^O and ^26^Mg artificially introduced into the contacted solution tend to enrich in the altered rim of a silicate sample with no observable sigmoid-shape diffusion profile toward the unaltered core^12^. Furthermore, oscillatory zonings are often observed in the altered rim of a silicate sample (Fig. 1A)^7^,^12^,^13^,^14^. Such oscillatory behaviors are difficult to reconcile with the traditional leached layer concept^7^.

The contradicting observations clearly indicate the complexity of silicate material dissolution and call for a new theory to account for such complexity. The new theory must also be able to explain the multiple stages of a glass dissolution process (Fig. 2A). As a silicate glass corrodes, the corrosion rate generally decreases, due to the reduction in chemical affinity for silicate network dissolution and the formation of a passivating layer on glass surfaces^3^,^4^,^6^. Interestingly, a sharp increase in corrosion rate after a long period of rate drop has been observed in silicate glass dissolution experiments^15^,^16^, posing a serious concern about our ability to predict the long-term performance of borosilicate glasses as a durable waste form for nuclear waste disposal. The underlying mechanism for this rate resumption remains unknown^4^.

## Results

Our research started with addressing the issue of oscillatory zonings. Oscillatory zonings on archeologic glass samples have been attributed to seasonal fluctuations in temperature or hydrologic conditions^17^. But this explanation is apparently not applicable to the oscillatory zonings produced in laboratory experiments, which are usually conducted under static conditions with no externally imposed periodic changes on experimental conditions^12^,^13^. Interestingly, temporal oscillations in silicate dissolution have been observed directly in laboratory experiments^18^,^19^,^20^. Thus, the observed oscillatory dissolution behaviors must be self-organizational^13^, i.e., they must originate from the internal dynamics of solid-water interactions. Self-organization requires a positive feedback among physical and chemical processes involved in a system^21^,^22^. In silicate dissolution, the following positive feedback may operate: As a silicate material corrodes, cations (notably Na^+^) in the material are released into the solution, resulting in a high cation concentration and pH at the reaction front (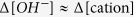 for charge balance). Under alkaline conditions, silicate dissolution is catalyzed by both hydroxyl groups and cations^23^,^24^,^25^. The resultant high pH and cation concentration enhance silicate material dissolution, which in turn accelerates cation release. A silicate dissolution rate usually has a V-shape dependence on pH (ref. 23). The proposed self-accelerating mechanism operates only under alkaline conditions, that is, on the right branch of the rate curve in Fig. 2B.

To test the concept, we formulated a nonlinear dynamic model for glass dissolution (see equations and nomenclatures in Methods). Numerical simulations of the model show that the proposed mechanism generates oscillatory dissolution within a reasonable parameter space (Fig. 3). The equilibrium silica concentration for glass dissolution is estimated to be ~10^−3^ to 10^−2^ M (ref. 26). The cation concentration at the isoelectric point (IEP) (*C*_*IEP*_) (Fig. 2B) can vary widely depending on experimental or environmental conditions; a range of 10^−4^ to 10^−2^ M could be a reasonable choice^18^. Accordingly, the concentration ratio between silica and cation (*θ*) in Fig. 3A varies from 1 to 100. Oscillatory dissolution occurs over a wide range of *θ* but only within a narrow range (0.8 to 3.0) of *γ*. The narrow *γ* range implies that self-organization requires the dissolution rate and the mass exchange rate to be on the same order of magnitude so that the two processes can interplay with each other. At the beginning of the dissolution, no altered zone is developed and the dissolution process is overwhelmed by mass exchange. Thus, *γ* always starts from a low value, and then increases as the alteration product builds up, leading to a transition from a plain altered zone to an oscillatory zone (Fig. 4A), as observed in leaching experiments^12^,^13^. Oscillatory zoning thus tends to emerge at a late stage of a dissolution process. Self-organization also requires the order of the dissolution reaction with respect to cation to be higher than 1.4 (Fig. 4B). The dissolution rate is known to be proportional to [≡*OH*^−^]^2^ (refs 27,28), where ≡*OH*^−^ is the adsorbed hydroxyls. At a low surface coverage, [≡*OH*^−^]∝[*OH*^−^]. Considering the additional catalytic effect of cations through ionic strength^25^, the reaction order *n* is estimated to be ~1.0 to 2.5. Parameter *α* is constrained between 0.3 and 0.6 from glass compositions (refs 9,12,19). Oscillatory dissolution is thus expected to be relatively common in silicate material corrosion (Fig. 4).

The time scale for each oscillation (*T*_*b*_) is estimated to be (See methods):


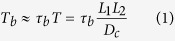


where *τ*_*b*_ is the scaled time for each oscillation, estimated to be 10–50 from the numerical simulations (Fig. 3). A gap of micrometers has been observed between the pristine glass surface and the altered rim^13^. If we take this gap as the boundary layer, the thicknesses of the boundary layer (*L*_1_) would be on the order of ~1 to 10 μm. The thickness of the altered zone (*L*_2_) is estimated to be on the order of ~10 to 100 μm, based on microstructural analyses of leached silicate samples (refs 12,13). The altered zone thickness is expected to be bounded by the thickness of the plain altered zone formed prior to oscillatory dissolution and the thickness of the overall altered zone. The diffusion coefficient (*D*_*c*_) for a mobile species in the altered zone in a reorganized dense silica gel could be as low as 6 × 10^−15^ cm^2^/s (ref. 6). As discussed below, such a dense layer is unlikely to form under the conditions for oscillatory dissolution. The actual altered zone could be porous^13^ with a much higher diffusion coefficient. *D*_*c*_ is thus chosen to be 10^−13^ to 10^−11^ cm^2^/s. From Equation (1), the time scale for each oscillation is estimated to range from hours to years, consistent with observations^12^,^13^,^17^. It is interesting to note that an archeologic study of ancient Roman glass artifacts show that each oscillatory layer might have formed over a time scale of a year^17^. Similarly, the thickness of each band (*L*_*b*_) can be estimated by (see Methods):


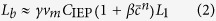


where 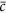 is a typical scaled cation concentration chosen to be ~10 based on the numerical simulations; and *v*_*m*_ is the volume of pristine solid containing 1 mole of SiO_2_, estimated to be ~30 cm^3^/mole^29^. For *γ* ≈ 1.0, *β* ≈ 5 (Figs 3 and 4) and *C*_*IEP*_ = 10^−4^ to 10^−2^ M (see the discussion above), *L*_*b*_ is estimated to range from 0.1*L*_1_ to a few *L*_1_ units, that is, sub-micrometers to tens of micrometers, consistent with observations^10^,^13^.

The proposed mechanism provides a logical explanation for the occurrence of leached layers. The evolution of the leached layer thickness (*L*_*g*_) is governed by (see Methods for nomenclatures):


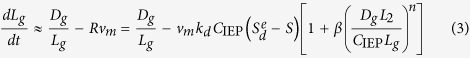


where *D*_*g*_ is the cation diffusion coefficient in the leached layer. As material dissolution pushes the chemistry of the boundary layer toward the right branch of the dissolution curve in Fig. 2B and the reaction product builds up (increasing *L*_*2*_), the second term in the far right-hand side would eventually overtake the first term. For reaction order *n* > 1, the leached layer then becomes progressively thinner (i.e., a smaller *L*_*g*_ makes the *L*_*g*_ decrease even faster)–a self-sharpening mechanism for the formation of an extremely sharp interface between a pristine silicate material and the surrounding altered rim^10^,^13^. Leached layers are thus transient and tend to form at an early stage of a dissolution process under mildly acid to neutral conditions (Fig. 2B).

The stage of alteration resumption is also a natural consequence of the self-accelerating mechanism. Assume that glass dissolution starts on the lower part of the dissolution curve in Fig. 2B. Due to a low dissolution rate, a leached layer forms. As the dissolution proceeds, the dissolution rate increases as more cations accumulate in the boundary layer, leading to disappearance of the leached layer. When the dissolution rate becomes on the same order of magnitude as the mass exchange rate with the bulk solution, oscillatory dissolution may emerge. Eventually, the dissolution rate overtakes the mass exchange rate, leading to a “runaway” situation with a sharp increase in the cation concentration at the interface and therefore the dissolution rate. The sharp increase in both cation concentration and pH inevitably causes zeolite precipitation (Fig. S1). Contradicting the existing view that the zeolite precipitation causes alteration resumption^26^, our work suggests that zeolite formation is a consequence of the alteration resumption process, consistent with experimental observations that adding initial amount of zeolite (analcime) had no effect on glass alteration^30^. The precipitation of zeolite would eventually limit further increase in the reaction rate by removing cations from the boundary layer (Fig. S1). Thus, the resumption rate may represent a long-term rate for silicate glass dissolution. Whether or how soon the alteration resumption occurs apparently depends on glass composition as well as the chemistry of the contacted solution. Thus, the durability of a glass can be improved by choosing an appropriate glass composition such that a proper alteration product will form which will limit the dissolution to the lower part of the dissolution curve (Fig. 2B). Therefore, in principle, it is possible to formulate a glass composition that can adapt to a specific environmental condition to achieve optimal performance in terms of glass durability.

## Discussion

One aspect to be considered in glass formulation is the content of dissolvable anionic components such as boron in a borosilicate glass. The release of boric acid from glass dissolution tends to compensate the aforementioned positive feedback between cation release and cation-enhanced dissolution kinetics. This effect can be factored into our model through an effective cation concentration [effective cation], defined as [total dissolved cations]–[total dissolved anions] with proper charge corrections. In this case,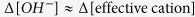. The release of dissolved anionic species would thus push the dissolution curve in Fig. 2B toward the right and therefore delay the transition of dissolution stages. In this sense, the inclusion of boron component in a glass formulation may enhance the durability of the glass. Similarly, any component from the contacted solution that can change the pH at the dissolution front through a reaction should also be taken into account. For instance, in glass alteration in seawater, Mg^2+^ from seawater can react with glass components to form Mg-smectite^31^:


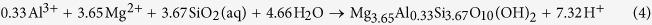


The H^+^ produced from this reaction tends to counter the pH rise caused by cation release from glass dissolution. Smectite precipitation may thus play the same role as zeolite precipitation in limiting the dissolution rate “runaway” at the alteration resumption stage (Fig. 2B). It should be pointed out that the model presented here is only a first-order approximation to an actual complex dissolution process. A detailed evaluation of the potential impacts of material composition and water chemistry on dissolution kinetics requires an explicit consideration of chemical speciation and secondary mineral formation, which is beyond the scope of this paper but certainly an interesting topic for future research.

Temperature can play an important role in controlling the transition from one dissolution mode to another. The activation energy for aqueous ion diffusion typically ranges from 15 to 20 kJ/mole (ref. 32) whereas the activation energy for glass dissolution is ~86 kJ/mole (ref. 31). Assume that glass dissolution starts at the lower part of the dissolution curve in Fig. 2B. As the temperature increases, the dissolution rate accelerates faster than the diffusion rate and eventually overtakes the latter, leading to oscillatory dissolution and possibly alteration rate resumption (Fig. 2B). Thus, increasing temperature may enhance the chance for oscillatory zoning and alteration resumption. This may be a reason why the most extensive set of oscillatory zoning was observed in the experiment conducted at an elevated temperature (150 °C)^12^. Interestingly, alteration resumption has not been observed for borosilicate glasses in laboratory experiments at pH < 10 and temperature <90 ^o^C (ref. 30). In general, based on our model, a low temperature, high dissolvable anion content, or low solution pH tends to delay or inhibit the occurrence of oscillatory zoning and alteration rate resumption.

This proposed self-accelerating mechanism may cause the instability of a dissolution front: A higher dissolution rate at one surface location would lead to a higher accumulation of cations and therefore a higher dissolution rate at that location, which would in turn promote the local dissolution. This positive feedback may lead to the formation of a wavy dissolution front^22^. Interestingly, such wavy fronts are observed in silicate glass corrosion^13^. The development of a wavy alteration front coupled with the oscillatory zoning may be responsible for the formation of growth rings on the surface of corroded Roman glasses^33^. Growth rings are concentric banding patterns with a typical size of ~1 mm; the patterns are roughly equally spaced with each consisting of ~190 concentric rings in average; and those patterns seem to have grown simultaneously since the interface between two neighboring patterns is a straight line^13^. Based on the concept proposed here, growth rings are thus the geometric intercepts of wavy repetitive alteration zones with a view plane.

Finally, the proposed mechanism provides a new perspective for predicting silicate mineral weathering rates in natural systems. The pH and cation concentration of water at a dissolution interface could be much higher than those in the bulk solution (e.g. extractable pore water). Using bulk pore water chemistry for prediction, as done currently, may significantly underestimate mineral reaction rates. Similarly, silicate mineral weathering in environments with limited water availability, for example, in semiarid to arid regions, may be more dynamic than previously thought. The proposed mechanism can create local high pH microenvironments in rocks and thus enhance CO_2_ dissolution and mineralization, a mechanism probably responsible for continuous large CO_2_ uptake by desert soils^33^.

## Methods

**Figure 1:**
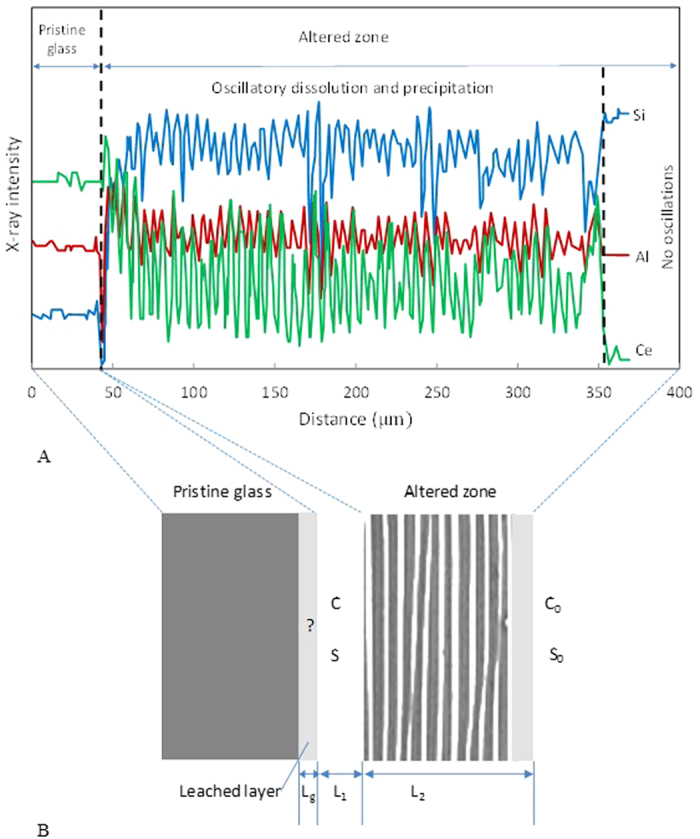
Oscillatory borosilicate glass dissolution and mineral precipitation as indicated by compositional zoning in an alteration zone (A) and schematic representation of modeling system (B). The actual Al and Ce contents are in the ranges of 0–0.4** **wt% and 0–2.4 wt%, respectively^12^. Data in (**A**) were taken from ref. 12.

The modeling system for oscillatory silicate glass dissolution is shown in Fig. 1B. It consists of three physical domains: a pristine glass domain, a boundary layer, and an alteration zone. The leached layer in the figure is not included, because it is likely to be absent in oscillatory dissolution due to the self-sharpening effect of a reaction front as discussed above. The dynamics of aqueous silicate material dissolution can be described by:


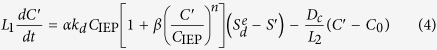



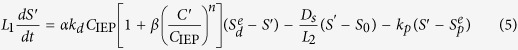


where

*C′* – Cation concentration within the boundary layer

*C*_0_ – Cation concentration in the bulk solution (outside the altered zone)

*D*_c_ – Diffusion coefficient of cations in the altered zone

*D*_*s*_ – Diffusion coefficient of dissolved silica in the altered zone

*L*_1_ – Thickness of the boundary layer at the dissolution interface

*L*_2_ – Thickness of the altered zone

*k*_*d*_ – Reaction rate constant for silicate material dissolution

*k*_*p*_ – Reaction rate constant for silica mineral precipitation

*n* – Order of silicate dissolution reaction with respect to cation

*S′* – Silica concentration within the boundary layer

*S*_0_ – Silica concentration in the bulk solution


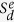
 – Equilibrium silica concentration for material dissolution


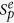
 – Equilibrium silica concentration for silica precipitation

*t* – Time

*α* – Molar ratio of cations (mainly Na^+^) to Si^4+^ in the pristine silicate material

*β* – Positive constant characterizing the catalytic effect of cations on silicate material dissolution

**Figure 2:**
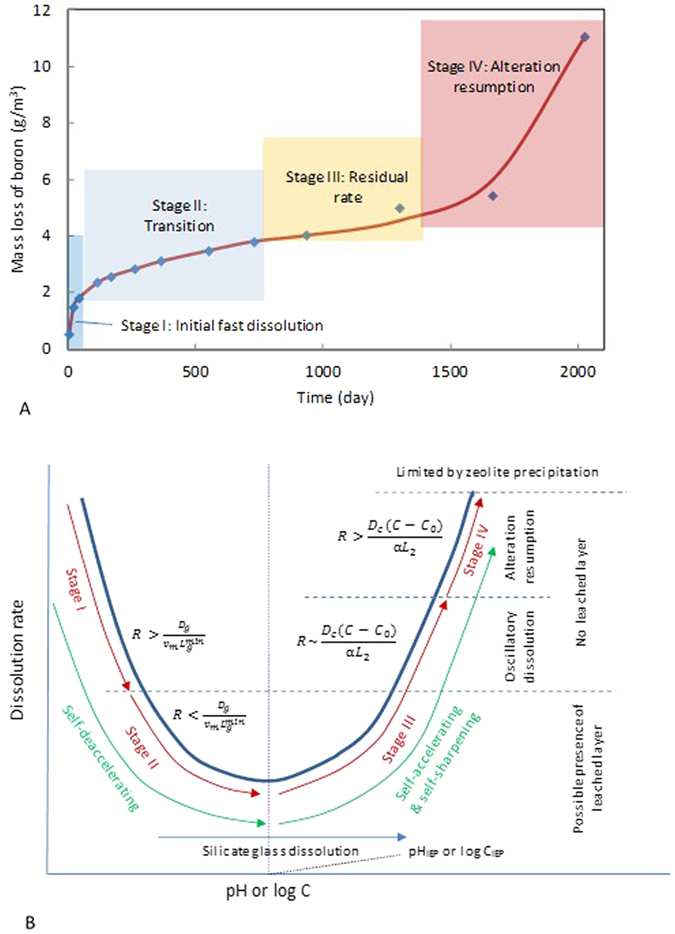
Evolution of silicate glass dissolution. (**A**) and its nonlinear dynamics (**B**). A positive feedback between glass dissolution and solution chemistry may lead to oscillatory glass dissolution, interface sharpening and alteration resumption at the late stage of glass corrosion. Note that this feedback becomes effective only for the base leg of the dissolution curve (**B**). IEP: Isoelectric point. 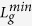 is the minimum spatial resolution for a microanalysis and imaging technique for characterizing the sharpness of a reaction front (≤1 nm). The data points in (**A**) were taken from ref. 15.

**Figure 3:**
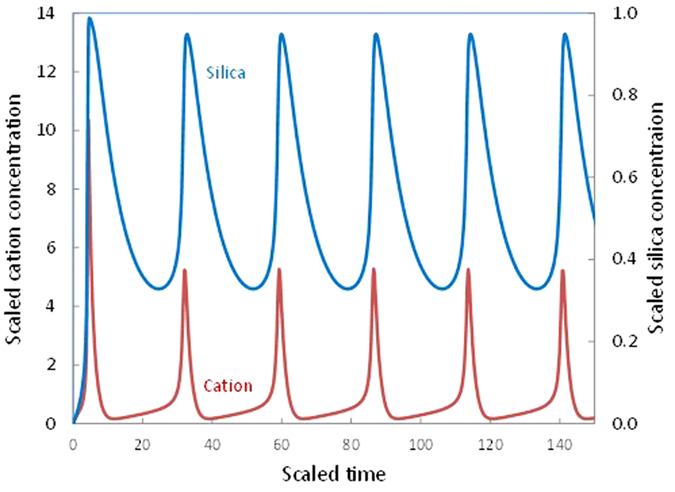
Predicted concentration oscillations at the glass dissolution interface. Parameter values used in the simulation: α = 0.4, γ = 0.9, n = 2.0, β = 5.0, θ = 40, η = 2, κ = 2.0, c_0_ = s_0_ = 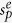 = 0.001 (see Methods for parameter definitions). Silica precipitation rate and therefore the Si content in the altered zone oscillates in phase with the dissolved silica concentration [Equation (5)]. The amounts of impurities incorporated into the precipitated silica are expected to be proportional to both the concentrations of the impurities (roughly mimicked by the cation concentration shown in the figure) and the silica precipitation rate^22^. The proposed model thus predicts that impurity contents in an altered zone of a silicate material oscillate precisely in phase with each other but slightly out of phase with silica content, as observed^12^ (Fig. 1A).

The first terms on the right-hand side of Equations (4) and (5) represent the mass accumulation due to silicate material dissolution. The factor 
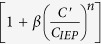
 is introduced to capture the catalytic effect of cations on the dissolution of silicate network in the materials. We here only consider silicate material dissolution under alkaline conditions, that is, on the right branch of the dissolution curve in Fig. 2B. The dissolution reaction is assumed to be first order with respect to the dissolved silica concentration^26^. The other terms on the right-hand side of the equations represent the mass exchange between the boundary layer and the bulk solution or the mass consumption by silica mineral precipitation.

Equations (4) and (5) can then be cast into the following dimensionless equations:


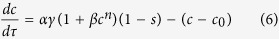



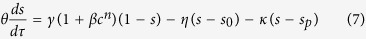


with the following scaling factors:


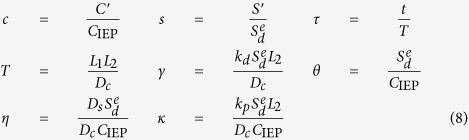


where

c - Scaled silica concentration

s – Scaled silica concentrations

*T* – Characteristic time scale of the system

*γ* – Scaled glass dissolution rate relative to the rate of diffusional mass exchange between the boundary layer and the bulk solution

*η* – Scaled diffusivity ratio between silica and cation

*θ* – Characteristic concentration ratio between silica and cation

*κ* – Scaled rate constant for silica mineral precipitation

*τ* – Scaled time

**Figure 4:**
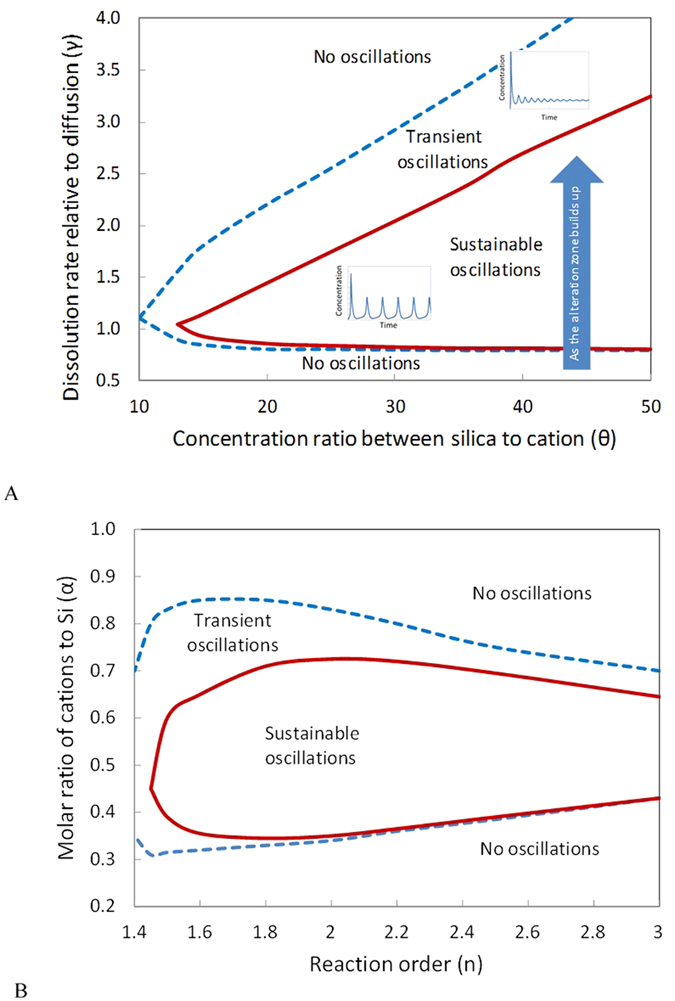
Behavior diagrams for silicate glass dissolution. As the alteration zone builds up, the dissolution transits from a no-oscillation state to an oscillation state as observed^12^. Parameter values used in the calculation: (**A**) α = 0.4, n = 2.0, β = 0.9, η = 2, κ = 2.0, c_0_ = s_0_ = 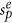 = 0.001; (**B**) γ = 1.0, n = 2.0, β = 1.0, θ = 40, κ = 2.0, c_0_ = s_0_ = 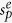 = 0.001 (see Methods for parameter definitions).

Equations (6) and (7) were solved using an arbitrary-precision ordinary differential equation solver from python package mpmath ( http://mpmath.org/) and Mathematica (Wolfram Research Inc. 2015). The behavior diagrams in Fig. 4 were constructed by numerical simulations.

## Additional Information

**How to cite this article**: Wang, Y. *et al.* Nonlinear dynamics and instability of aqueous dissolution of silicate glasses and minerals. *Sci. Rep.* **6**, 30256; doi: 10.1038/srep30256 (2016).

## Supplementary Material

Supplementary Information
