# Supplementary material for: Nonlinear dynamics and instability of aqueous dissolution of silicate glasses and minerals
Source: Sci Rep. 2016 Jul 22;6:30256. doi: 10.1038/srep30256 (PMC4957211; doi:10.1038/srep30256)
Supplement: Supplementary Information [file srep30256-s1.pdf]

Supplementary information

Nonlinear dynamics and instability of aqueous dissolution of silicate glasses and minerals

Yifeng Wang[1]*, Carlos F. Jove-Colon[1] and Kristopher L. Kuhlman[1]

[1]Sandia National Laboratories, P. O. Box 5800, Albuquerque, New Mexico 87185-0779, USA
E-mail: ywang@sandia.gov

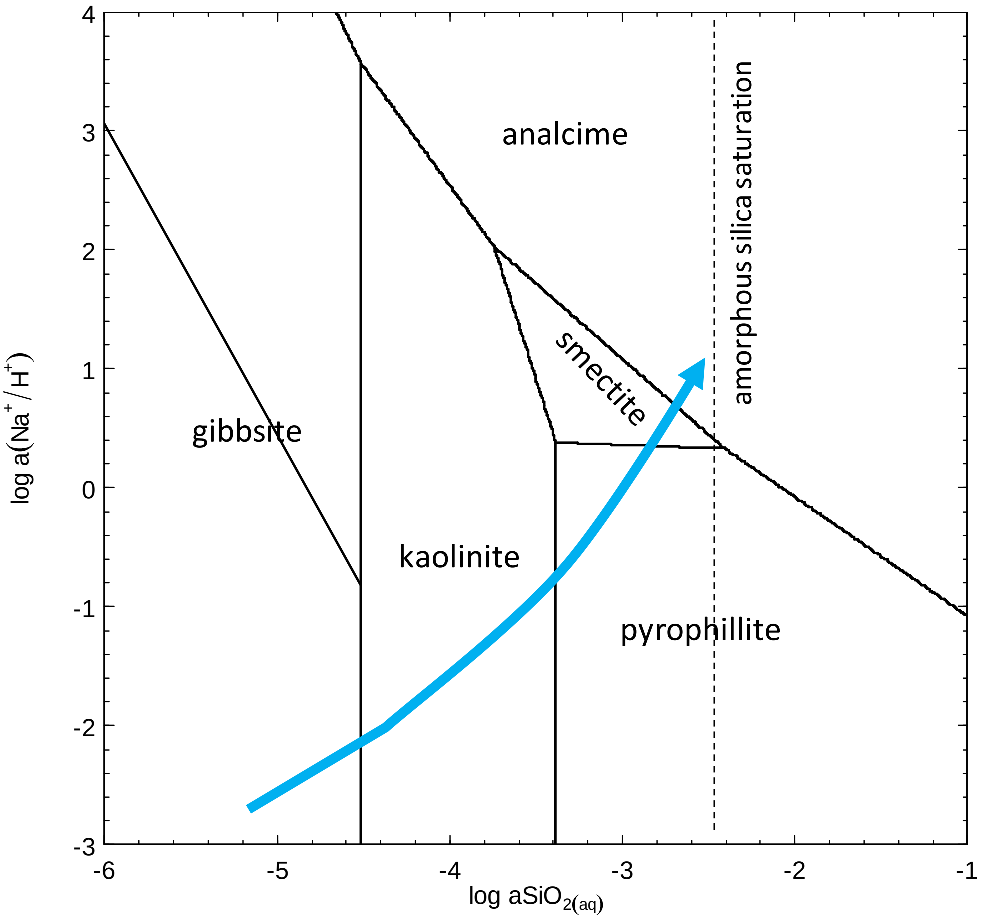

Figure S1| Evolution of water chemistry and mineral precipitation at a reaction front of silicate material dissolution. Sources of thermodynamic data: Kaolinite - Blanc, P., A. Lassin, P. Piantone, M. Azaroual, N. Jacquemet, A. Fabbri, and A. Gaucher, Thermoddem: A geochemical database focused on low temperature water/rock interactions and waste materials. Applied Geochemistry, 2012. 27: p. 2107-2116. Smectite MX80 - Blanc, P., A. Lassin, P. Piantone, M. Azaroual, N. Jacquemet, A. Fabbri, and A. Gaucher, Thermoddem: A geochemical database focused on low temperature water/rock interactions and

waste materials. Applied Geochemistry, 2012. 27: p. 2107-2116. Gibbsite - (1) Tutolo, B.M., X.-Z. Kong, W.E. Seyfried, Jr., and M.O. Saar, Internal consistency in aqueous geochemical data revisited: Applications to the aluminum system. Geochim. Cosmochim. Acta, 2014. 133: p. 216-234. Or Robie, R.A. and B.S. Hemingway, Thermodynamic properties of minerals and related substances at 298.15 K and 1 bar (10^5 pascals) pressure and at higher temperatures. U S Geological Survey bulletin 2131, 461 pp. Analcime - Neuhoff, P.S., G.L. Hovis, G. Balassone, and J.F. Stebbins, Thermodynamic properties of analcime solid solutions. Am. J. Sci., 2004. 304(1): p. 21-66. Pyrophillite: Tutolo, B.M., X.-Z. Kong, W.E. Seyfried, Jr., and M.O. Saar, Internal consistency in aqueous geochemical data revisited: Applications to the aluminum system. Geochim. Cosmochim. Acta, 2014. 133: p. 216-234.
